# Pattern of buprenorphine abuse among opioid abusers in Nepal

**DOI:** 10.4103/0019-5545.70978

**Published:** 2010

**Authors:** Tapas Kumar Aich, Manoj Dhungana, Roshija Khanal

**Affiliations:** Department of Psychiatry, Universal College of Medical Sciences, Bhairahawa, Nepal

**Keywords:** Buprenorphine abuse, IDU, Nepal, opioid abuse

## Abstract

**Background:**

Although buprenorphine abusers are a common clinical entity, literature on them is rare in Nepal.

**Aim:**

To assess whether injectable opioid abusers are any different a subgroup vis-a-vis brown sugar abusers in relation to their demographic and clinical profiles.

**Materials and Methods:**

Seventy-six opioid abusers, who were admitted over a period of one year, in our de-addiction center, were included in the present study. They were divided into two groups based on the history of the presence or absence of buprenorphine injection abuse in them. The demographic and clinical profiles of these two groups were studied and compared.

**Results:**

The most characteristic opioid abuse pattern was the abuse of brown sugar through inhalation (chasing). A total of 32 (42.1%) among them had a history of injectable drug abuse (IDU). Most characteristic buprenorphine abuse pattern seen was an evolution from injectable buprenorphine to triple injection to brown sugar abuse (Reverse Transition). Injection buprenorphine abusers, who attended our clinic, were older in age and had a history of a longer duration of abuse than their counterparts who abused opioid drugs through the inhalational route only. Their lifetime diagnosis revealed a polysubstance abuse pattern. They were more unstable, impulsive, and disorganized in their behavior pattern, suggestive of the presence of inadequate personality traits. There were high instances of injection-related side effects in the form of the presence of thrombophlebitis, HIV positivity, and clinical AIDS in them.

**Conclusion:**

Findings of the current research indicate the presence of a subgroup of patient population among opioid abusers with a history of injectable buprenorphine abuse, with characteristic personality traits, pattern of drug abuse, and associated physical complications resulting from it.

## INTRODUCTION

Buprenorphine is a mixed agonist antagonist opioid, available in sublingual/oral and parenteral form. Beginning in 1983, a number of reports have highlighted the abuse of buprenorphine in oral as well as parenteral forms, and alone as well as in combination with benzodiazepines or antihistamines.[1–4] The route of administration is an important characteristic related to abuse of drugs, and 29–50% of opioid (heroin)-dependent patients change their route of administration over time.[5–9]

According to the published data, the first case of buprenorphine abuse in India was registered in 1987.[10] To date more than a dozen reports have been published in India covering various aspects of buprenorphine abuse.[11–13] Of late, the parenteral use of buprenorphine, mostly in combination with benzodiazepines and antihistaminics, has shown an increased trend.[14–16]

In Nepal by the mid ‘70s cases of drug dependency on opiates, especially heroin began to appear. Both official and unofficial estimates indicate that from virtually no cases in 1976, there were over 10,000 and even as high as 20,000 heroin addicts a decade later, concentrated in the Kathmandu Valley.[17,18] In an ethnographic study on the drug use in Nepal, Jutkowitz *et al*. reported detailed clinical characteristics of 16 heroin abusers.[18] Many of them were polydrug abusers, but there was no report of injectable buprenorphine abuse. The present account is a modest effort to express our clinical experience in dealing with these patients in a tertiary teaching hospital set up.

## MATERIALS AND METHODS

It was a clinical study spanning over a period of two years (January 2003–December 2004). The study set up was the de-addiction center of the Universal College of Medical Sciences (UCMS), at Bhairahawa, Nepal. Ethical approval for the study was taken from the institute’s ‘Ethics Approval Committee’ prior to starting the study. Individual patient’s consent was taken before including their clinical data in the present study. Inclusion criteria were that all inpatients were within the age range of 15–45 years with a DSM-IV diagnosis of opioid abuse/dependence.[19] Exclusion criteria were the history of opioid abuse secondary to functional/organic mental illness and mental retardation.

All the cases that were admitted during the stated period, with a diagnosis of opioid abuse/dependence and/or polysubstance abuse with predominantly opioid dependence were included for initial assessment. Patients were re-interviewed and reassessed in the ward, with history clarified from the patients as well as patient’s guardians (wherever present), and the diagnoses were confirmed according to DSM-IV. The DSM-IV questionnaire was used to note down the pattern of drug abuse during the last five years (lifetime diagnosis) and during the last one month (current diagnosis).

All these subjects were administered a semi-structured questionnaire to collect information on the demographic data, duration of drugs being used and types of opiates used, onset and duration of injection used, and reasons for shift from inhalational to injectable route (Transition) and from injectable to inhalational route (reverse transition), if any. A separate questionnaire was prepared for injectable drug abusers (IDUs) noting the pattern of abuse, triple injection abuse, IDU-related side effects, HIV and AIDS status, and so on. A total of 76 opioid abusers were included in the present study, and were admitted and treated during the first year of the study period (January 2003–December 2003), and who later fulfilled our inclusion and exclusion criteria. These patients were followed up for a maximum period of one year (till December 2004), to observe the pattern of abstinence, relapse/recurrence or readmission following use/abuse of drugs. Simple descriptive statistics such as frequency and percentages, chi-square test, and t-test were used to analyze the data thus available. For carrying out a detailed statistical analyses, a computer-assisted statistical series (Statistical Program for Social Sciences: SPSS version 10.0) was sought.

## RESULTS

Few demographic and clinical profiles of treatment seekers in our center are revealed in Table 1. They were in their early twenties at the time of their first contact with us. They mostly (93.3%) came from a nearby locality of 50 kilometer radius. A majority of them were accompanied by their parents/spouse or close relatives, who were actively involved in the treatment process. A majority of the patients in our study belonged to the lower and middle socioeconomic background. Interestingly, none of the subjects of our study were female.

**Table 1:** Demographic and clinical profiles (N=76)

Variable	
Age in years (mean age)	25.4 (4.6)
Religion	
Hindu	73 (96.1%)
Muslim	3 (3.9%)
Locality	
Within city area	41 (53.9%)
within 50 km.	30 (39.5%)
50–200 km.	4 (5.3%)
Beyond 200 km.	1 (1.3%)
First contact	
Alone/groups	16 (21%)
with guardians/spouse	60 (79%)
Lifetime diagnosis	
Opioid abuse / dependence	18 (23.7%)
Polysubstance abuse	58 (76.3%)
Current diagnosis	
Opioid abuse / dependence	50 (65.8%)
Polysubstance abuse	26 (34.2%)
Pattern of abuse	
Injection drug Use (IDU)	32 (42.1%)
Abuse by inhalation	44 (57.9%)

Eighteen patients (23.7%) fulfilled the diagnosis of pure opioid dependence, whereas, the remaining 58 (76.3%) patients were polysubstance abusers, abusing at least three or more number of substances during the last five years. When we analyzed the abuse pattern during the last one month (Current diagnosis) we found that 68.8% (50) of them were abusing only the opioid group of drugs. A total of 32 among 76 (42.1%) patients had a history of buprenorphine abuse suggestive of IDU either as a current abuse pattern or with a history of IDU in the past.

Table 2compares the clinical profiles of two groups: one with a history of Injectable drug use (IDU) and another of patients with a history of pure inhalation drug users. Injection drug users had significantly high lifetime history of polysubstance abuse (*P*<0.05). Their mean age and duration of abuse was significantly more than their counterparts (*P*<0.001). They also showed significantly inadequate personality traits (*P*<0.05).

**Table 2:** Clinical profiles compared

Variable	IDU inhalation	Abuse	t/Χ^2^	P
Lifetime diagnosis				
Opioid abuse	4	14	Χ^2^=3.8	0.04*
Polysubstance abuse	28	30		
Current diagnosis				
Opioid abuse	19	31	Χ^2^=1.01	0.2
Polysubstance abuse	13	13		
Age (in years)				
Mean (SD)	27.5 (4.4)	23.8(4.1)	t=3.8	0.000***
Duration of abuse (in years)				
Mean (SD)	4.2 (2.9)	2.3 (1.7)	t=3.7	0.000***
Period of hospitalization (in days)				
Mean (SD)	8.8 (4.9)	10.1 (7.7)	t=0.7	0.4
Follow-up				
None	20	29	Χ^2^=0.05	0.9
Once	5	8		
Twice or more	6	8		
Readmission				
Yes	3	6	Χ^2^=0.2	0.6
No	28	39		
Inadequate personality traits				
Present	22	10	Χ^2^=4.1	0.04*
Absent	10	24		

* Significant at 0.05 level

*** Significant at 0.001 level

Although a commonly reported phenomena was the transition from brown sugar abuse to IDU, our study group had a higher percentage of reverse transition pattern of abuse (21.1% cases) or a pattern fluctuating to-and-fro from brown sugar to IDU to brown sugar [Table 3]. More than 10% of the cases also had a history of triple injection abuse. More than 70% of the patients with injection drug abuse had superficial and/or deep thrombophlebitis with seven of them diagnosed as being HIV positive by ELISA testing [Table 3]. Although we could not go for Western Blot or other sophisticated testing to confirm AIDS, at least three of them had clinical AIDS according to the WHO criteria.

**Table 3:** Pattern of evolution of opioid abuse (N=76) and IDU related physical complications (*n*=32)

	No. of patients (%)
Pattern of abuse (N=76)	
Transition: Brown sugar →idu	6 (7.9%)
Reverse Transition: idu→Brown sugar	16 (21.1%)
Brown sugar →idu → Brown sugar	5 (6.6%)
Brown sugar only	45 (59.2%)
Combined Brown sugar and idu	2 (2.6%)
Idu only	2 (2.6%)
Inj. Brown sugar	1 (1.3%)
Triple inj. (Buprenorphine+Diazepam+Promethazine)	8 (10.5%)
IDU related physical complications (n=32)	
Thrombophlebitis	23 (71.8%)
HIV positive	7 (21.8%)
AIDS	3 (9.3%)
Cellulitis, Absces	1 (3.1%)

## DISCUSSION

The typical profile of a buprenorphine abuser that has been reported commonly in the literature is that of an urban, young to middle aged (19–42 years) male, who has had some school or college education, and has a low-to-middle level occupation.[10,11,13,20] Our patients also had a similar demographic profile. A majority of treatment seekers (79%) in our study group were coerced into the treatment plan by their guardians/spouse. This, perhaps indicates a lower level of motivation among our patients, also evidenced by the fact that there was a high dropout rate during follow-up.

A majority of patients attending our center followed the ‘royal road to opioid’: from nicotine via alcohol, cough syrup, cannabis, sedatives, to opioid abuse. Before graduating to pure opioid abuse, many of these patients went through abuse of milder varieties of the opioid group of drugs, especially phensedyl cough syrup, spasmoproxyvon capsules, and the like. The most characteristic opioid abuse pattern was the abuse of brown sugar through inhalation (chasing). Thirty-two (42.1%) patients had a history of injection buprenorphine abuse (IDU) either as a current abuse pattern or with a history of IDU in the past [Table 3]. Abuse of buprenorphine alone was reported in 13–54% of the cases, by various authors, in the past.[12,20–22]

Analyzing their lifetime diagnosis pattern, we found that many of our patients (76.3%) went back and forth between opioid abuse and other substances of abuse, depending on the availability of brown sugar/buprenorphine and the availability of the necessary finance. Cough syrup (Phensedyl), nitrazepam (Nitrosun), and alcohol were the common add-on drugs to opioid abuse, for these patients. Other reported co-abused substances were Cannabis and Carisoprodol.[10–20]

The most characteristic buprenorphine abuse pattern seen in our center was an evolution from injection buprenorphine (Tidigesic inj.) to triple injection to brown sugar abuse (Reverse transition). Available literature in this regard points to the contrary. Most reported cases had graduated to buprenorphine abuse from the abuse of another opioid, heroin, in up to 84% of the cases, which had preceded the usual period of three to five years and a maximum of 2–12 years.[11,20,22,23] Research reported that the main reasons for a shift to the parenteral route (transition) were related to, self-treatment of heroin dependence with injection of buprenorphine, low cost of buprenorphine, and non-availability of heroin/brown sugar. The reasons for different patterns of evolution of opioid abuse in our center were probably due to the easy availability of buprenorphine injection across the Indo-Nepal border and the frequent use of injection by the peer group.

The common pattern of co-abuse was an intravenous cocktail of 0.6 mg of buprenorphine with 10 mg of diazepam, and 100 mg of promethazine, taken two to four times daily. This pattern of co-abuse as an intravenous cocktail has already been reported by various authors in the past.[10,20] The outcome of the long duration of injection drug abuse in this patient population was that there were acute and chronic infections, cellulitis and abscess, and thrombophlebitis. The HIV test was positive in 22.6% (7) of the cases and clinical AIDS was diagnosed in 9.7% (3) of the cases [Table 3].

That parenteral administration of drugs is associated with the risk of HIV infection, hepatitis B, infections such as cellulitis, abscesses, and endocarditis has already been reported in the past.[24] A higher prevalence of high-risk behaviors like unprotected sex, sex with multiple partners, in commercial sex workers, homosexuality, and inadequate cleaning and sharing of injection material with multiple partners, have all been reported for buprenorphine abusers.[25] Injectable drug abusers who attended our clinic were significantly older in age (*P*<0.001) and had a longer duration of drug abuse history than brown sugar abusers who used the inhalational route only (*P*<0.001) [Table 2]. Lifetime diagnosis of polysubstance abuse was significantly more common among them (*P*<0.05) and they showed a significantly higher socially disruptive behavior, suggestive of unstable, inadequate personality traits (*P*<0.05) [Table 2]. This instability in personality was reflected in more cases of abuse/suspected abuse in the ward, more cases of absconding from the ward, more instances of breaking hospital rules and regulations and confrontation with hospital staff.

## CONCLUSION

Very few articles are available till date which tried to analyse the pattern of buprenorphine abuse among opioid abusers in Nepal.[26]. Findings of the current research probably indicate the presence of a subgroup of patient population among opioid abusers. This subgroup of injection drug users was older in age and had a history of a longer duration of abuse than opioid abusers, with no history of injection drug abuse. Their lifetime diagnosis revealed a polysubstance abuse pattern. They were more unstable, impulsive, and disorganized in their behavior pattern than their counterparts, who abused the drug through the inhalational route only. There were high instances of injection-related side effects in them, in the form of, thrombophlebitis, HIV positivity, and clinical AIDS.

Non-availability of laboratory tests to confirm drug use/abuse and poor follow-up may be envisaged as the main drawback of the present study. We need to have a wider-based study with more patient population to come to a definite conclusion and generalize the findings of the present study.

## References

[1] Strang J (1985). Abuse of buprenorphine. Lancet.

[2] O’Connor JJ, Moloney E, Travers R, Campbell A (1988). Buprenorphine abuse among opiate addicts. Br J Addict.

[3] Hammerseley R, Lavelle T, Forsyth A (1990). Buprenorphine and temazepam abuse. Br J Addict.

[4] Harper I (1883). Temgesic abuse. N Z Med J.

[5] Strang J, Griffiths P, Powis B, Abbey J, Gossop M (1997). How constant is an individual’s route of heroin administration. Data from treatment and nontreatment samples?. Drug Alcohol Depend.

[6] Griffiths P, Gossop M, Powis B, Strang J (1992). Extent and nature of transitions of route among heroin addicts in treatment preliminary data from the Drug Transition study (UK Transition Report). Br J Addict.

[7] De la Fuente L, Barrio G, Royuela L, Bravo MJ (1997). The transition from injecting to smoking heroin in three Spanish cities. The Spanish Group for the Study of the Route of Heroin Administration. Addiction.

[8] Swift W, Maher L, Sunjic S (1999). Transitions between routes of heroin administration: A study of Caucasian and Indochinese heroin users in south western Sydney, Australia. Addiction.

[9] Griffiths P, Gossop M, Powis B, Strang J (1994). Transitions in patterns of heroin administration: A study of heroin chasers and heroin injectors. Addiction.

[10] Singh RA, Mattoo SK, Malhotra A, Varma VK (1992). Cases of buprenorphine abuse in India. Acta Psychiatr Scand.

[11] Basu D, Varma VK, Malhotra AK (1990). Buprenorphine dependence: A new addiction in India. Disabil Impair.

[12] Chowdhury AN, Chowdhury S (1990). Buprenorphine abuse: Report from India. Br J Addict.

[13] Nizamie SH, Sharma LN (1990). Buprenorphine abuse: A case report. Indian J Psychiatry.

[14] Ray R, Ray R (1998). Current extent and pattern of drug abuse. South Asia Drug Demand Reduction Report.

[15] Chavan BS, Tripathi BM, Lal R (1995). Outcome of parenteral buprenorphine abuse. Indian J Psychiatry.

[16] Basu D, Mattoo SK, Malhotra A, Gupta N, Malhotra R (2000). A longitudinal study of male addicts attending an addiction clinic in India. Addiction.

[17] Bhandari B, Subba C (1992). Students and Drugs in Nepal.

[18] Jutkowitz JM, Spielmann H, Koehler U, Lohani J, Pande A (1997). Drug use in Nepal: The view from the street. Subst Use Misuse.

[19] (1994). American Psychiatric Assocation (APA). Diagnostic and Statistical Manual of Mental Disorders, (DSMIV).

[20] Sharma Y, Mattoo SK (1999). Buprenorphine abuse in India an update. Indian J Psychiatry.

[21] Gupta DK, Desai NG, Chandiramani K (1996). Pattern of multiple substance use in heroin dependent individuals. Indian J Psychiatry.

[22] Vinay K, Singh BK (1996). Reasons behind increasing buprenorphine abuse. Indian J Psychiatry.

[23] Umesh Babu SB, Chaturvedi SK (1995). Changing patterns of opiate abuse with a focus on buprenorphine. Indian J Psychiatry.

[24] Ghodse H, Ghodse H (1989). Complications of drug abuse and their treatment. Drugs and addictive behavioural guide to treatment.

[25] Malhotra A, Balaji M, Basu D, Mattoo SK, Varma VK, Sehgal S (1993). HIV screening and risk behaviour in psychoactive substance users. Indian J Med Res.

[26] Ojha SP, Pokhrel P, Acharya RP, Pandey KR, Bhusal CL, Marhatta MN (2002). Socio-psychological study among injectable drug users in Kathmandu valley. J Nepal Med Assoc.

